# Home video review in a pediatric clinical neurophysiology led multidisciplinary team meeting

**DOI:** 10.1002/epd2.20310

**Published:** 2024-12-16

**Authors:** Aneirin Rhys‐Potter, Maria Cunha, Matthew Sparkes, Zaloa Agirre‐Arrizubieta, Sushma Goyal, Joel S. Winston

**Affiliations:** ^1^ GKT School of Medicine London UK; ^2^ Paediatric EEG Department, Evelina London Children's Hospital Guy's & St Thomas' NHS Foundation Trust London UK; ^3^ Department of Clinical Neurophysiology King's College Hospital NHS Foundation Trust London UK; ^4^ Department of Basic and Clinical Neurosciences, Institute of Psychiatry, Psychology and Neuroscience King's College London London UK

**Keywords:** home video review (HVR), infantile spasms, pediatric epilepsy, triage, video‐EEG telemetry (VT)

## Abstract

**Objective:**

Assessment of the value of review of home videos by a pediatric multidisciplinary team (MDT) in a pediatric neurophysiology department.

**Methods:**

We describe and evaluate the review of home videos alongside clinical history and previous investigations from patients referred to the Evelina pediatric EEG department at a twice‐monthly MDT meeting between 01/2021 and 09/2022. We retrospectively analyzed measures of video quality, quantity and duration, time taken from referral to MDT meeting, pre‐MDT and post‐MDT meeting proposed diagnosis and clinical outcomes. Feedback from referring doctors was obtained by a survey.

**Results:**

There were 36 referrals for 34 patients totalling 123 videos. There was a median delay of 10 days between video upload and final report. After the MDT meeting the number of referrals classified as uncertain fell from 15 to 2. The number of referrals classified as non‐epileptic events increased from seven to 18. The number classified as infantile spasms fell from six to two. Overall, 26 of 36 referrals had a change in diagnosis and 26 of 36 referrals were triaged away from the video‐EEG telemetry (VT) waiting list. Nine out of ten referring doctors reported that MDT discussion improved their understanding of the events.

**Significance:**

HVR is a useful tool that has been successfully incorporated into our neurophysiology department's workflow. These early results suggest benefits from adopting an MDT meeting may include an early diagnosis and management approach based upon consensus. Using HVR in a pediatric population may help triage urgent cases, conserve specialized neurophysiological investigations and streamline workflows to improve the efficiency of pediatric referrals.

**Key points**

- Patient and carer home videos are a valuable potential source of clinical information about paroxysmal events in children.
- New online platforms allow secure upload, review and storage of patient and carer home videos.
- A multidisciplinary team review of such videos can help to guide and optimize patient management.

## 1 INTRODUCTION

Home videos are potentially a useful adjunct to the clinical history and neurophysiological tests for diagnosing and monitoring seizures. These videos can be as good as, or superior to, clinical history alone at predicting video EEG diagnosis.^1^, ^2^, ^3^, ^4^, ^5^ In children, supplementing a clinical history with phone videos has been shown to increase accurate diagnoses by an average of 3.9% in epileptic seizures and 11.5% in non‐epileptic events^5^.

In 2020, Evelina London Children's Hospital pediatric neurosciences department enabled parents, carers and clinicians to upload videos to a National Health Service (NHS) approved cloud database.^6^ Prior to this there was no dedicated, secure portal for videos. Videos were uploaded for a variety of indications and a total of 2% of those uploaded were referred to clinical neurophysiology after being reviewed by the requesting clinician.

Clinical neurophysiology services in the UK have no currently defined pathway or clinical code for review of standalone home videos in the absence of an EEG.^7^ Our pediatric EEG department already provided a twice monthly EEG multidisciplinary team (MDT) meeting during which the on‐call team and other pediatric neuroscience colleagues could discuss their patients' EEGs. This became the Home Video Review (HVR) meeting, in which the referrer had to be present to provide clinical details and, if available, previous EEG data was also reviewed. Here we report our initial experiences and evaluation of this service.

## 2 METHODS

**FIGURE 1:**
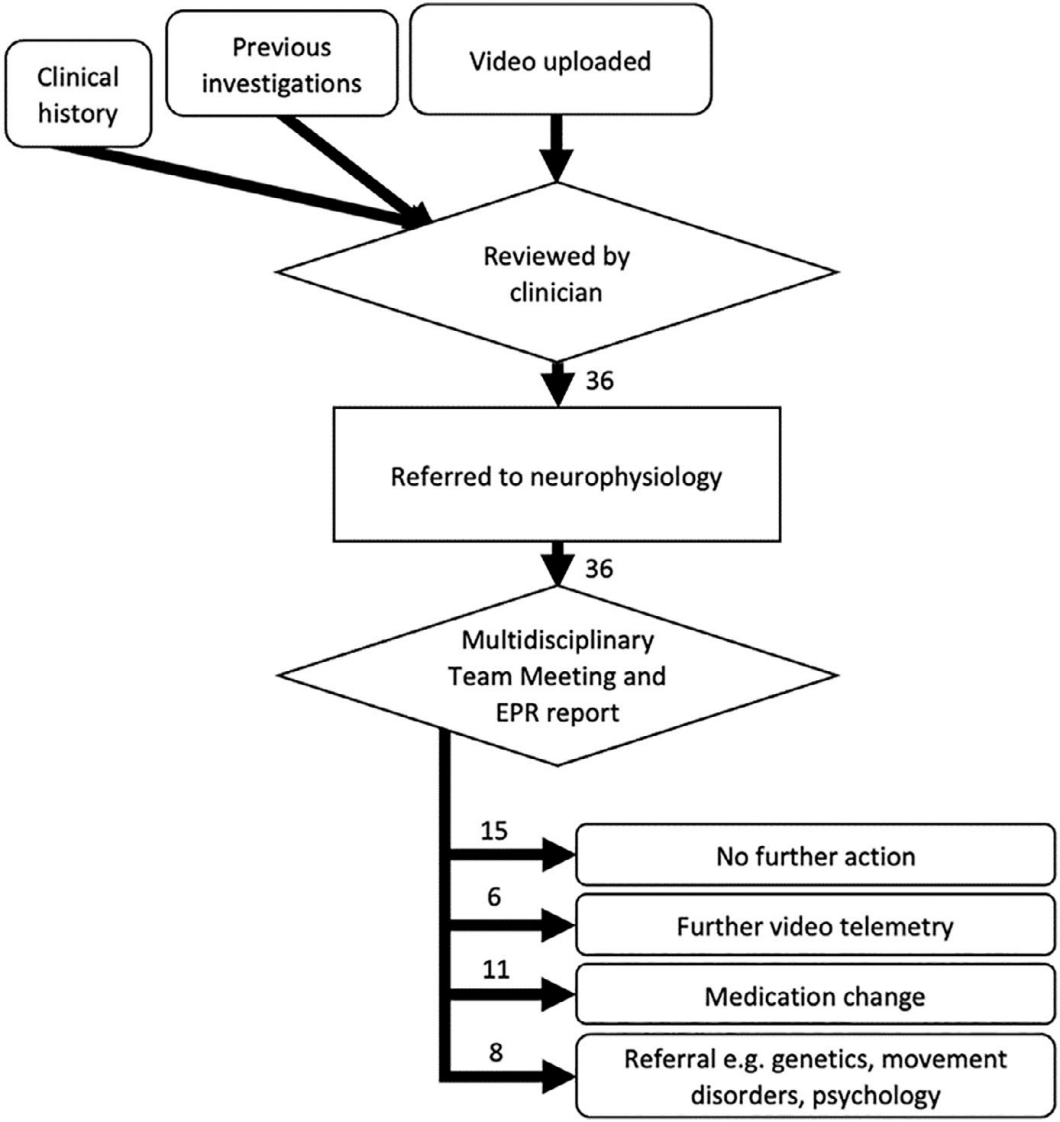
Flowchart of video uploads, referrals and MDT outcomes. Numbers represent raw counts.

Our MDT meeting was attended by clinical neurophysiologists, the referring doctor, pediatric neurologists including those with an expertise in epilepsy and movement disorders, sleep and behavioral disorders, other pediatricians, EEG technicians and epilepsy nurses. Trainees could also attend. Presentation included clinical history, previous investigations and playing the home video. Through verbal consensus the MDT meeting provided an initial clinical classification of the event and made a recommendation for further investigations or referrals if necessary (Figure 1). A report was then completed by the clinical neurophysiology service, uploaded to the patient's electronic healthcare record and sent to the referring doctor.

Data were collated from HVRs at MDTs between 01/01/2021 and 01/09/2022. Data included measures of video quality (see below), quantity (number of videos) and duration, time from referral to MDT meeting, pre‐ and post‐MDT meeting proposed diagnosis and clinical outcomes. Video quality was categorized as “very good” or “good” (sufficient for diagnosis) or “poor” (insufficient for diagnosis). Further video features were categorized including whether the relevant anatomical features were in view, whether the start of an event was captured and whether there was interaction with the patient. A survey of referring doctors was conducted to gather feedback including measures of the time spent on initial video review, how the MDT meeting changed the management of the patient as well as allowing free text feedback on the HVR meetings. An internal database review was conducted in June 2024 to look for re‐referrals of patients previously discussed at HVR meetings.

Permission for video upload and sharing amongst relevant medical staff was sought automatically via the cloud database vCreate Neuro <https://www.vcreate.tv>. Patients or their parents/guardians could withdraw their videos at any time. The current study was conducted as an evaluation of the clinical service and was registered locally without requirement for written consent from patients or their careers.

## 3 RESULTS

### 3.1 Patients and pathway

We analyzed results from 36 sequentially discussed referrals (15 female; median age 4 years; range: 10 weeks–15 years) from 34 patients. A total of 19 individual referring clinicians made referrals; nine were general pediatricians (typically with a special interest in epilepsy) and 10 were pediatric neurologists. Individual referrers referred between one and nine cases. Patients referred had a range of comorbidities:

- 11 of 36 referrals had genetic comorbidities
- 9 of 36 had a diagnosis of epilepsy
- 3 of 36 had a diagnosis of a movement disorder
- 2 of 36 had neonatal intracerebral hemorrhage
- 8 of 36 had radiological abnormalities
- 2 of 36 had developmental delay
- 4 of 36 had autism
- 2 of 36 had learning disability

There were a total of 123 home videos with a mean 3.4 videos/referral and mode of 1 video/referral. Video duration ranged from 0.2–11.4 min, with a mean of 2.0 min. Video picture quality was mostly considered sufficient for clinical assessment (classified “very good” [19/36] or “good” [16/36]). One referral had videos with poor picture quality as it was taken overnight by a home monitoring system.

**FIGURE 2:**
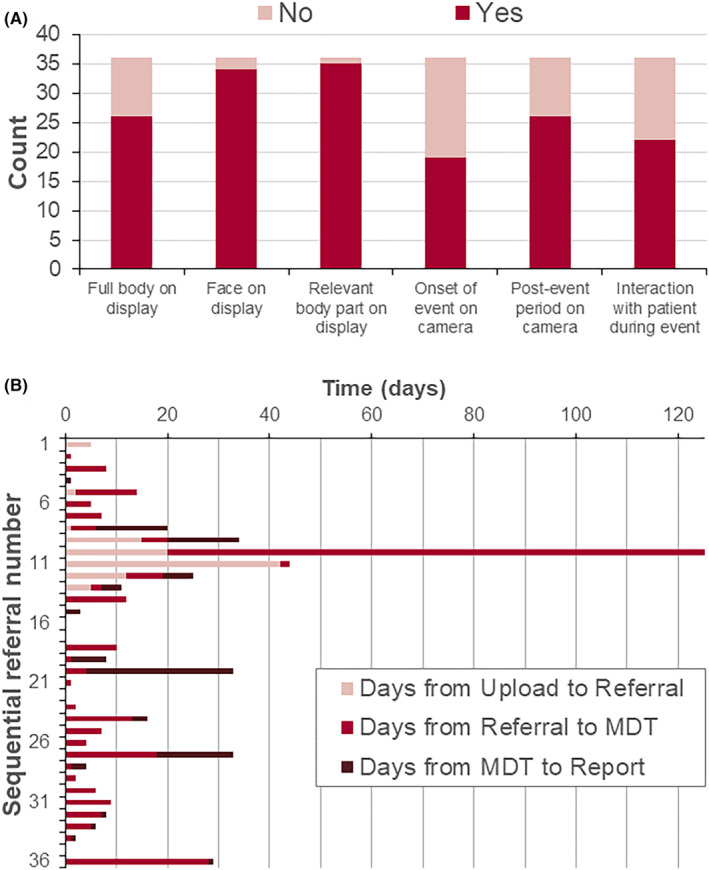
Measures of video and service quality. (A) Videos were assessed as to whether particular features of interest were captured; data are shown as presence/absence of each feature in raw counts. (B) Sequential referrals were audited for the time taken from video upload to MDT conclusion being issued with each phase measured separately.

35/36 referrals showed videos with the relevant body part displayed. The full body on display, face on display and post event period were shown in over 70% of referrals. Only 22/36 referrals showed interaction with the patient during the event. Of note, a high proportion of referrals (17/36) did not capture the start of the event, which often includes clinically relevant information (Figure 2A). However, a significant proportion of the events happened at predictable times or in clusters so parents/carers were able to capture the beginning of an event.

There was an overall median 10 days for video upload, referral, MDT meeting and issue of a final report (mean 19 days, range 0–123 days; Figure 2B). Most time was spent between initial video referral and MDT meeting (median 5 days). A common contribution to this delay was referring consultants finding it difficult to arrange attendance at the MDT meeting to present cases due to clashing clinical or professional commitments. One referral took 123 days due to such a delay in coordinating with the referring consultant.

Seven referrals had no record of previous EEGs. Nine referrals had previous Video EEG Telemetry (VT) recordings with habitual events recorded in six of these studies. Two VT studies were abnormal without showing seizures, three VT studies confirmed the presence of both epileptic seizures and non‐epileptic events, two recorded epileptic seizures only and in one study the child was confirmed to have a movement disorder. After HVR review, repeat telemetry was advised in one child with previously recorded Myoclonic absences; this was to confirm the reported increase in seizure frequency to help guide treatment escalation. In the other eight children with previous VT studies, repeat telemetry was avoided after HVR.

Twenty referrals had previous inter‐ictal EEGs, of these seven were normal studies. In one child with a normal EEG a habitual non‐epileptic event was recorded during the EEG. After the MDT meeting VT was advised in three children, all with abnormal inter‐ictal EEGs. In the first of these VT was advised urgently at the child's regional centre as HVR was consistent with infantile spasms. In the second child VT was undertaken to confirm epilepsy classification as HVR was consistent with a focal seizure. In the third child VT was advised in the future should the frequency increase of current infrequent events seen on HVR, given the background history of lissencephaly. The referring clinician concluded this was not needed after the review in the follow up clinic.

A total of five patients had subsequent EEGs against the consensus of the MDT. Four were for patients that the HVR concluded showed non‐epileptic events and no further tests were required. The follow‐up EEGs in all four of these cases did not change the diagnosis from that of the HVR MDT. A fifth child had known epilepsy and the HVR MDT thought the episodes were in‐keeping with his previous episodes which was confirmed by a subsequent EEG.

### 3.2 Events

**FIGURE 3:**
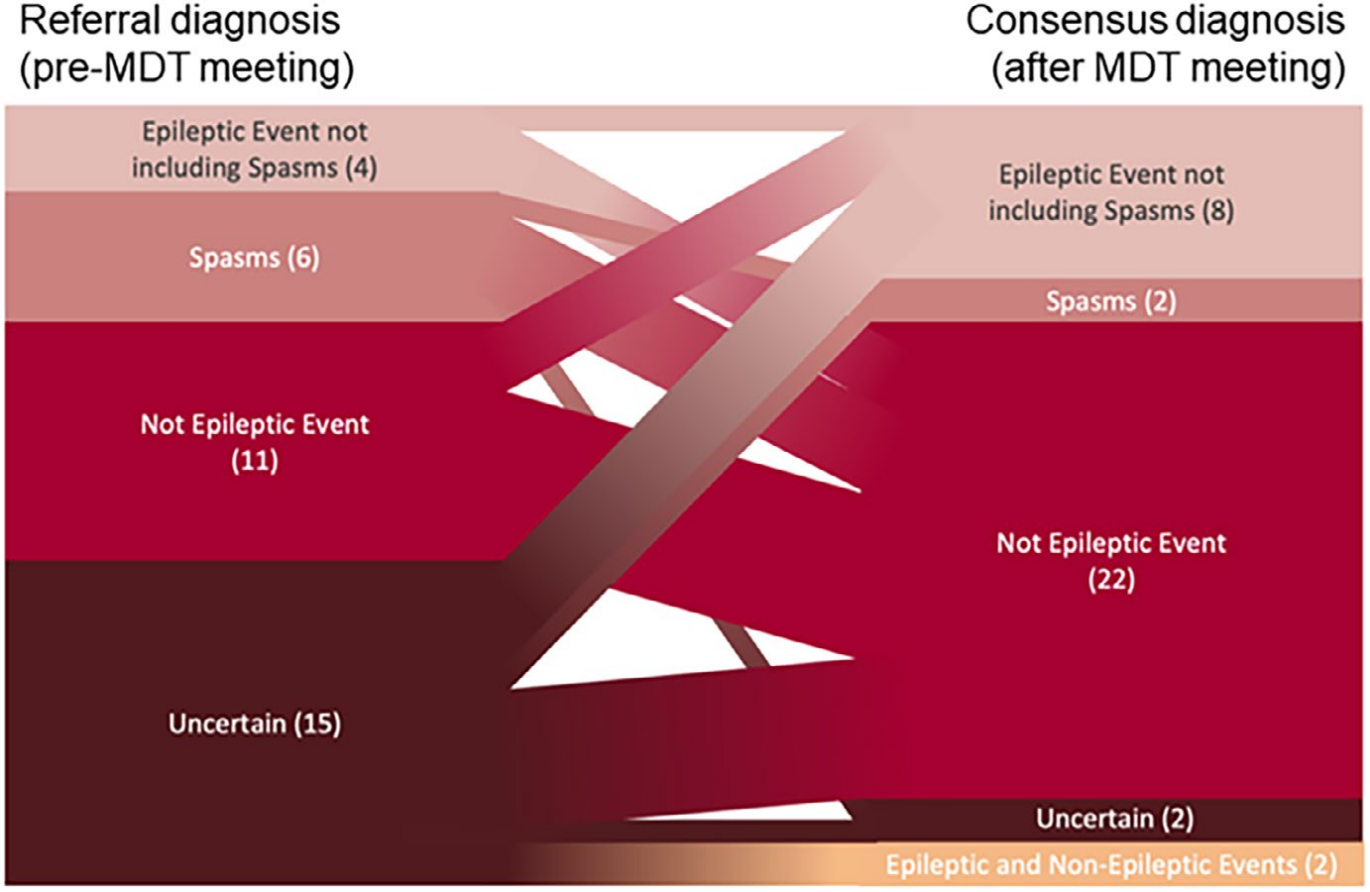
Sankey chart showing diagnostic flow from referral to the consensus diagnosis post‐MDT. Numbers in parentheses represent raw counts.

After the MDT meeting the number of referrals classified as non‐epileptic events increased from 11 to 22 (Figure 3). These included “transient infant movements”^8^ which comprised head shaking, shuddering attacks, self‐gratification or other types of previously reported complex stereotypies. It was helpful to triage these videos as they were sometimes referred urgently as “spasms”, with a few having already had previous EEGs but without habitual event capture. Of those children in whom HVR classified non‐epileptic events, three had episodes consistent with a diagnosis of functional neurological disorder (FND). Four of the non‐epileptic event referrals were suggestive of other neurological disorders and were followed up by the referring doctor without further neurophysiological tests required.

Ten children were classified as having convincing epileptic events of which two were spasms. This was a similar total proportion to before the meeting, but the number classified as “spasms” decreased from six to two. The first child had two videos that were classified as bilateral convulsive seizures and an absence that was similar in semiology to events previously recorded on video‐EEG telemetry (VT). The second had six videos with nocturnal events consistent with frontal lobe hypermotor seizures. Follow‐up MRI revealed a structural lesion. The third child's videos revealed events comparable to previously recorded seizures on VT and were classified as myoclonic absence seizures on MDT review. Two children had a mixture of epileptic and non‐epileptic events.

The number of referrals classified as uncertain fell from 15 to 2. Of the two for whom consensus MDT diagnosis was not reached, the first referral consisted of very brief eye movements in a child with developmental malformation and delay. A second video was requested but this was also too short at 11 s to arrive at a clinical decision. The second referral showed only one video (captured mid event) not typical of spasms but was classified as uncertain as there were 20 repeated movements. More videos were requested but not provided. Both patients ultimately had a VT study arranged after the MDT.

### 3.3 Outcomes

Twenty‐six patients referred to the service were triaged off the neurophysiology waiting list; of these, six avoided a repeat VT. Five patients were triaged off the neurophysiology waiting list but ultimately later had a further EEG which did not change the HVR diagnosis. Eleven referrals resulted in an attempt to optimize medication, while 25 had no change. Changes to one patient's medication may have been delayed by the MDT HVR as their initial videos were too brief (11 s) and were deemed inconclusive. An EEG 75 days after the first referral ultimately showed atonic and tonic seizures different in semiology to the videos originally sent and treatment was then optimized.

### 3.4 Feedback from referring consultants

Most consultants (7/10) estimated that they spent over 5 min analyzing the videos before requesting a referral to neurophysiology. Almost all consultants (9/10) reported that HVR improved their understanding of the event. 4/5 consultants responding to the question said that the HVR MDT modulated follow‐up investigations; *“my patient avoided unnecessary sleep investigations (polysomnography and MSLT) as well as unnecessary further neurophysiological tests e.g. telemetry*”. One respondent suggested that the mandatory attendance of the referring doctor at the HVR MDT meeting made the service inaccessible because they did not routinely work on the day it was held.

## 4 DISCUSSION

HVR is a useful tool in the triaging and diagnostic pathway which we have shown can be incorporated into a pediatric clinical neurophysiology service within the framework of an MDT meeting. Most videos supplied by parents are of sufficient quality for clinical interpretation (Figure 2A). Time from video upload to MDT review was generally relatively short (Figure 2B), and triage based upon referral information allows the most urgent cases to be seen earlier. Consultants referring to the service reported an improved understanding of the events with some improvements suggested. Further, a recent report from the Scottish Health Technologies Group showed “parents and carers were predominantly positive about the ease of use of vCreate Neuro, how ‘connected’ it made them feel to the clinical team”.^6^

The MDT meeting provided clinical consensus from a varied group of specialists including epilepsy, movement disorders and childhood behavioral disorders. MDT meetings have been shown to improve clinical decision making^9^, ^10^ which is potentially particularly valuable when reviewing videos of suspected seizures without the added benefit of the accompanying EEG.

We see the role of HVR as a triaging tool for patients who present with apparent new onset seizures or a new event type in the context of known epilepsy. Patients with previous seizure capture can have a comparison between the new home videos and previous recordings to determine if they are presenting with a new seizure type and require further investigations or management. This model uses the consultant neurophysiologist's expertise and access to previous telemetry videos to help referring clinicians prioritize patients for further neurophysiology investigations.

MDT‐based pediatric HVR could be especially helpful with some specific patient groups:

- Triaging urgent referrals for example, for possible infantile spasms
- Patients who have non‐epileptic events^11^
- Patients who have events too infrequent to offer a good likelihood of capture on VT^12^
- Patients who have had previous VT, but parents report a change in the nature of events
- Patients who are less likely to tolerate VT well, such as those with severe learning disabilities or autism^13^

HVR has also been used at our hospital for urgent triaging of EEG referrals of children presenting to the Emergency Department. Physicians uncertain about whether a patient needs to be referred for VT can either encourage parents to upload videos of events or can (with consent) film and upload events themselves. These cases can be seen for an initial prompt response by the attending clinical neurophysiologist and then later reviewed at the MDT meeting for clinical consensus and formal report. In our data this is particularly relevant to the cases of suspected infantile spasms where HVR showed 4/6 of the cases referred were more likely non‐epileptic events and did not require urgent EEGs. Urgent EEGs are particularly time and labor‐intensive as they require an EEG physiologist and doctor to be pulled from their normal daily workflow. Reducing the number of urgent referrals, such as possible infantile spasms, to the clinical neurophysiology service would increase the efficiency of both the emergency service and the department overall.

The HVR MDT can also provide educational opportunities to clinicians and students. As an online meeting multiple hospitals could be involved including centers without pediatric epilepsy neurophysiology, widening access to expert opinion. In this hospital it has given general pediatricians faster access to specialist epilepsy neurophysiology opinion without needing to go through pediatric neurology as expressed by some survey respondents.

### 4.1 Study limitations

A major limitation is imposed by the retrospective nature of the study without a gold‐standard investigation offered to all patients independent of the MDT outcome. This means that, for instance, where the MDT determined events as not epileptic in nature there was no routine follow‐up VT study to provide confirmation. All cases were followed up clinically by their referring team and review of electronic healthcare records did not provide any examples in which the MDT outcome was altered, including in five patients who eventually had further video‐telemetry despite the advice of the MDT that this was not indicated. Follow‐up was limited to what could be accessed via electronic healthcare records from our centre; given that this is the regional centre it is reasonable to expect that we cases for whom MDT outcomes were refuted by subsequent clinical progression would have returned. We cannot exclude that some patients might have moved out of the area or sought opinions or diagnosis elsewhere. A further limitation includes the fact that this was a study in an exclusively pediatric patient population. However, other studies have shown video review is reliable in adults^1^, ^2^, ^3^, ^4^, ^5^ and therefore suggests a similar HVR service could be adopted for adult patients. As with all MDTs there is no definitive or known optimum method of ensuring a consensus is reached that fully includes the opinions of all the specialists involved. In this case, the meeting chair's decision was the documented decision of the MDT but this was determined after an open discussion.

### 4.2 Further improvements to the HVR service

To further improve video quality, guidance will be given to parents on what information is most useful to capture on video, such as the whole body, the start and end of the event and interactions with the patient. This may also be paired with an exemplar video.

To improve accessibility, we are allowing the clinical summary to be presented by a clinician who is familiar with the patient if the responsible referring clinician is unavailable. Alternatively, flexibility in meeting timings, for example holding them on different days or times throughout the month, should maximize the opportunities for attendance. There would likely be significantly increased uptake of HVR in other hospitals if it could be coded as a stand‐alone diagnostic procedure as suggested by others^14^ in order that the activity is appropriately acknowledged and reimbursed where appropriate.

## 5 CONCLUSION

HVR is a useful tool that has been successfully incorporated into our neurophysiology department's workflow. Early results suggest that benefits include prompt diagnosis and treatment by better triaging patients. The improved efficiency of the neurophysiology triage saves clinician and patient time, likely reduces unnecessary tests and allows more urgent cases to be seen sooner.

## CONFLICT OF INTEREST STATEMENT

None of the authors has any conflict of interest to disclose.

**Test yourself**

1. Which of the following is not considered a “mimic” of infantile spasms?

- Childhood gratification
- Bengn tonic up gaze
- Shuddering attacks
- Benign myoclonus of infancy

2 Home video review in an MDT setting is most useful for:

- Triaging urgent EEG referrals
- Classifying seizures
- Replacing VT if events are very frequent
- Ordering sleep studies

3 Who should attend a home video review multidisciplinary team meeting?

- Referring paediatrician
- Paediatric neurologist
- Epilepsy monitoring/Video telemetry unit team
- All of the above

Answers

1. B ‐ Benign tonic up gaze

2. A ‐ Triaging urgent EEG referrals

3. D ‐ All of the above

## Data Availability

Derived data are available from the authors upon reasonable request. The reported data reflect a registered clinical service evaluation and did not require external ethical approval.
